# Development and validation of a model and nomogram for breast cancer diagnosis based on quantitative analysis of serum disease-specific haptoglobin N-glycosylation

**DOI:** 10.1186/s12967-024-05039-4

**Published:** 2024-04-04

**Authors:** Linrong Li, Yali Xu, Zhizhen Lai, Dan Li, Qiang Sun, Zhili Li, Yidong Zhou

**Affiliations:** 1 Department of Breast Surgery, Peking Union Medical College Hospital, Chinese Academy of Medical Sciences and Peking Union Medical College, No. 41 Damucang Hutong, Xicheng District, Beijing, 100032 China; 2 Department of Biophysics and Structural Biology, Institute of Basic Medical Sciences, Chinese Academy of Medical Sciences & School of Basic Medicine, Peking Union Medical College, No. 5 Dongdan San Tiao, Beijing, 100005 China

**Keywords:** Glycosylation, Breast cancer, Diagnosis, Model, Nomogram

## Abstract

**Background:**

A better diagnostic marker is in need to distinguish breast cancer from suspicious breast lesions. The abnormal glycosylation of haptoglobin has been documented to assist cancer diagnosis. This study aims to evaluate disease-specific haptoglobin (DSHp)-β N-glycosylation as a potential biomarker for breast cancer diagnosis.

**Methods:**

DSHp-β chains of 497 patients with suspicious breast lesions who underwent breast surgery were separated from serum immunoinflammatory-related protein complexes. DSHp-β N-glycosylation was quantified by mass spectrometric analysis. After missing data imputation and propensity score matching, patients were randomly assigned to the training set (n = 269) and validation set (n = 113). Logistic regression analysis was employed in model and nomogram construction. The diagnostic performance was analyzed with receiver operating characteristic and calibration curves.

**Results:**

95 N-glycopeptides at glycosylation sites N207/N211, N241, and N184 were identified in 235 patients with benign breast diseases and 262 patients with breast cancer. DSHp-β N-tetrafucosyl and hexafucosyl were significantly increased in breast cancer compared with benign diseases (*p* < 0.001 and *p* = 0.001, respectively). The new diagnostic model and nomogram included GN2F2, G6N3F6, GN2FS at N184, G-N&G2S2, G2&G3NFS, G2N3F, GN3 at N207/N211, CEA, CA153, and could reliably distinguish breast cancer from benign diseases. For the training set, validation set, and training and validation sets, the area under the curves (AUCs) were 0.80 (95% CI: 0.75–0.86, specificity: 87%, sensitivity: 62%), 0.77 (95% CI:0.69–0.86, specificity: 75%, sensitivity: 69%), and 0.80 (95% CI:0.76–0.84, specificity: 77%, sensitivity: 68%), respectively. CEA, CA153, and their combination yielded AUCs of 0.62 (95% CI: 0.56–0.67, specificity: 29%, sensitivity: 90%), 0.65 (95% CI: 0.60–0.71, specificity: 74%, sensitivity: 51%), and 0.67 (95% CI: 0.62–0.73, specificity: 60%, sensitivity: 68%), respectively.

**Conclusions:**

The combination of DSHp-β N-glycopeptides, CEA, and CA153 might be a better serologic marker to differentiate between breast cancer and benign breast diseases. The dysregulated N-glycosylation of serum DSHp-β could provide insights into breast tumorigenesis.

**Supplementary Information:**

The online version contains supplementary material available at 10.1186/s12967-024-05039-4.

## Introduction

Breast cancer surpassed lung cancer as the most commonly diagnosed cancer since 2020. Over 2.2 million new cases and 685,000 deaths from breast cancer occurred globally that year [1]. It was estimated that in 2040, over 2.9 million new cases of breast cancer were to occur worldwide [2]. Besides, of all cancers, breast cancer was estimated to incur the third largest economic cost from 2020 to 2050, accounting for 7.7% of the global cost of cancers [3].

Early detection, diagnosis and treatment are promoted by the WHO’s Global Breast Cancer Initiative launched in Feb 2023. For now, mammography and ultrasound remain primary options for breast cancer early diagnosis, which is considered to reduce mortality and financial burden [4, 5]. Despite technological advancements, the radiographic evidence for breast cancer diagnosis was largely limited by a modest diagnostic performance and relative high cost. Our center, Peking Union Medical College Hospital (PUMCH, Beijing, China), recently reported that the ultrasound and mammography yielded area under the curves (AUCs) of 76.8% and 71.3%, respectively, to distinguish patients with breast cancer from those with suspicious breast lesions [6]. As for serum tumor markers, cancer antigen (CA) 153, CA27.29, and carcinoembryonic antigen (CEA) are widely used for monitoring of recurrence and treatment response [7], as approved by the Food and Drug Administration. However, their applications in the diagnostic settings were limited due to insufficient accuracy [8, 9].

Haptoglobin (Hp), as an acute phase glycoprotein, has been increasingly studied for tumorigenesis. Hp has four N-glycosylation sites on the β-chain, located at Asn 207, Asn 211, Asn 241, and Asn 184 [10]. Primarily produced in the liver, Hp exerts its functional role by binding with free hemoglobin in the process of intravascular or extravascular hemolysis, while the aberrant expression of Hp was frequently studied in infectious and non-infectious diseases, including cancer [11]. Interestingly, Hp-β was shown to be significantly upregulated in patients with breast cancer, compared to those with ovarian cancer and healthy controls [12, 13]. In breast cancer, the upregulation of Hp contributed to tumorigenesis through glycolytic activity modulation [12]. Meanwhile, the pre-diagnostic serum level of Hp was positively related to the risk of early death from breast cancer [14]. Notably, the abnormal glycosylation of Hp, as well as its potential application for cancer diagnosis and prognosis, has been well documented in prostate, colon, liver, lung, cervix, uterus, and ovary cancers [15–19]. However, the role of the glycosylation of Hp in breast cancer remains elusive [20].

In this study, we sought to delineate the glycosylation patterns of disease-specific Hp (DSHp) in an effort to assist differential diagnosis of breast cancer. MALDI-FTICR MS (matrix-assisted laser desorption/ionization-Fourier transform ion cyclotron resonance mass spectrometer) was used to gain quantitative data of DSHp-β N-glycosylation in 497 patients with suspicious breast lesions. For the first time, we constructed and validated a new model and nomogram to reliably and efficiently distinguish breast cancer from benign breast diseases based on 7 DSHp-β N-glycopeptides combined with 2 tumor markers.

## Materials and methods

### Sample collection

From Apr 2020 to Dec 2020, we enrolled 523 women who were hospitalized to PUMCH for surgery because of suspicious breast lesion. The inclusion criteria were: (1) female of any age; (2) had at least one breast lesion suspected of malignancy, either detected by physical examination or imaging; (3) signed informed consent. The exclusion criteria were: (1) underwent no surgery due to change of mind or pre-operative assessment; (2) metastatic or unresectable tumor. 1 mL of serum was collected together with routine blood tests from each patient before surgery, and was immediately frozen and stored at − 80 °C until mass spectrometric analysis. The patients were asked to fast for at least 8 h before the blood tests.

Results of the routine blood tests, including tumor markers (i.e., CEA, CA125, CA153), complete blood count, and blood biochemical markers (i.e., alanine transaminase, ALT; aspartate aminotransferase, AST; cholinesterase, ChE; total cholesterol, TC; triglyceride, TG; high-density lipoprotein cholesterol, HDLC; low-density lipoprotein cholesterol, LDLC; apolipoprotein A1, ApoA1; apolipoprotein B, ApoB; lipoprotein a, Lpa; free fatty acids, FFA; high-sensitivity C-reactive protein, hsCRP; glucose, Glu), were retrieved from the hospital information system (HIS). Tumor markers, complete blood count, and blood biochemical markers were measured at the Department of Clinical Laboratory of PUMCH using Roche Cobas E601 electrochemical luminescence analyzer (Hoffmann-La Roche AG., Basel, Switzerland), Sysmex XN2000 Automated Hematology analyzer (Sysmex, Kobe, Japan), and Beckman-Coulter AU 5800 (Beckman Coulter, Brea, USA), respectively. Other clinical characteristics of patients, including age at diagnosis and body mass index (BMI), were collected with the HIS. Pathological characteristics of each patient, including tumor classification, grade of differentiation, molecular subtype, and pathological stage were determined by referring to patients’ pathological reports of surgical specimens with the HIS in accordance with WHO Classification of Tumors of the breast and the National Comprehensive Cancer Network Guidelines [21, 22].

### Sample preparation

Serum immunoinflammatory-related protein complexes (IIRPCs) of each patient were isolated from 10 μL of serum by native-polyacrylamide gel electrophoresis (PAGE), and classified into different IIRPC patterns based on gel bands, as previously defined [18, 23]. The gel bands for each sample were cut and washed with ultrapure water, then reacted with 200 μL of 0.2 M dithiothreitol (Sigma-Aldrich, St. Louis, MO, USA) for 45 min at 37 °C, followed by 200 μL of 0.5 M iodoacetamide (Sigma-Aldrich, St. Louis, MO, USA) for 45 min at 37 °C. After washed with ultrapure water, the DSHp-β chains were separated from the gel bands by sodium dodecyl-sulfate (SDS)-PAGE. Then, the gel bands were cut into pieces and put into 96-well plates, with each well containing gel for one patient. Destaining and dehydration of the DSHp-β chains were conducted using 50% acetonitrile (ACN, Fisher Scientific, Fair Lawn, USA) and 100% ACN, respectively. Each sample was incubated at 37 °C overnight with 10 μL of 12.5 ng/μL sequencing grade modified trypsin (Promega, Madison, USA).

After that, the supernatant was aspirated and vacuum-freeze-dried. The DSHp-β N-glycopeptides were then enriched as previously described [18, 23]. Enrichment solution was prepared by dispersing 20 mg of Fe3O4@PANI in 10 mL of 80% ACN. 100 μL enrichment solution was added to each well of the 96-well plate. After shaken at 80 r/min for one hour, the supernatant was removed with magnetic separation. 80% ACN was used to wash out possible residual peptides. Next, we incubated the samples with 0.025% ammonia solution for 40 min at 37 °C to elute glycopeptides. Finally, the supernatant was collected and lyophilized for mass spectrometric analysis.

### Mass spectrometric analysis

Each prepared samples were dissolved in 5 μL of ultrapure water, from which 0.5 uL was spotted onto a MTP 384 AnchorChip target plate with transponder technology (Bruker Daltonics, Billerica, MA), and mixed with 0.5 uL of matrix solution containing 10 mg/ml α-cyano-4-hydroxycinnamic acid in 50% ACN with 0.1% trifluoroacetic acid (Fisher Scientific, Fair Lawn, USA). The detection of DSHp-β N-glycopeptides was conducted with 7.0 T Solarix XR MALDI-FTICR MS (Bruker Daltonics, Billerica, MA). Calibration was performed across *m/z* ranged 2000–7000, yielding a resolution of 490,000 at *m/z* 400 in the positive ion mode. We used polypeptide mix for calibration, which contained somatostatin_28, ky_37, dy_40, gp_52, ADRM and sl_61 at *m/z* 3147.4710, 3901.8705, 4328.1557, 5206.5147, 5969.9330, and 6814.5702, respectively. The mass spectra were acquired by 30 Avg Scan, with smart beam-II laser at 355 nm and 1,000 Hz frequency. An 1000-μm random walk width was used with 200 shots per scan. GlycoMod (https://web.expasy.org/glycomod/) was used for glycan structure prediction. The research team was unaware of the patients' pathology when collecting samples and conducting experiments.

### Statistical analysis

The *m/z* values of the detected glycopeptides with a signal to noise threshold of > 1.0 were saved with Microsoft Excel version 16.75.2 (Microsoft Corporation, Redmond, WA, USA), together with the clinicopathological characteristics of the patients. The missing *m/z* values were imputed with the half-minimal value of each sample. To fill in the missing values of clinical characteristics, we utilized multiple imputation with classification and regression trees as the conditional models (Additional file 1: Figs. S1, S2). After the outliers were manually adjusted, propensity score matching (PSM) was employed to balance potential confounding clinical variables, including age, BMI, complete blood count, and blood biochemical markers, between benign breast diseases and breast cancer.

The potential correlations of the DSHp-β glycopeptides and tumor markers were analyzed with Spearman's correlation analysis. Sequential modified Bonferroni correction was applied to control the false discovery rate. A two-sided p-value < 0.05 was considered statistically significant. Continuous variables were compared using Mann–Whitney U (Wilcoxon) tests, while categorical variables were compared using Pearson’s chi-squared tests. Differential analysis was performed on DSHp-β glycopeptides between benign breast diseases and breast cancer. The statistically significant changes of DSHp-β glycopeptides were selected using the criteria of a Bonferroni correction p-value of < 0.05 and absolute log2 Fold-change of > 0.195.

For construction and validation of the breast cancer diagnostic model, the PSM cohort was randomly divided into a training set and a validation set in a 7:3 ratio. Binary logistic regression was conducted on the training cohort to select variables from clinical characteristics and laboratory results for model construction. Forward stepwise regression was applied in the procedure to reduce multicollinearity, and variance inflation factor (VIF) was calculated for each variable in the final model. In order to evaluate diagnostic performance of the model, the receiver operating characteristic (ROC) curves and calibration curves were drawn for each cohort. The AUC, specificity, and sensitivity (adopted by the largest Youden's J statistic) of the model were calculated, with a nomogram constructed based on the model.

Data was processed using RStudio (R version 4.3.1). Imputation, PSM, differential analysis, and logistic regression were carried out using the mice, MatchIt, DESeq2, and rms packages, respectively. R packages VIM, ggplot2, corrplot, pheatmap, ggpubr, regplot, and pROC were used for visualization. A diagram of the study was created with BioRender.com.

## Results

### Patient characteristics

**Fig. 1:**
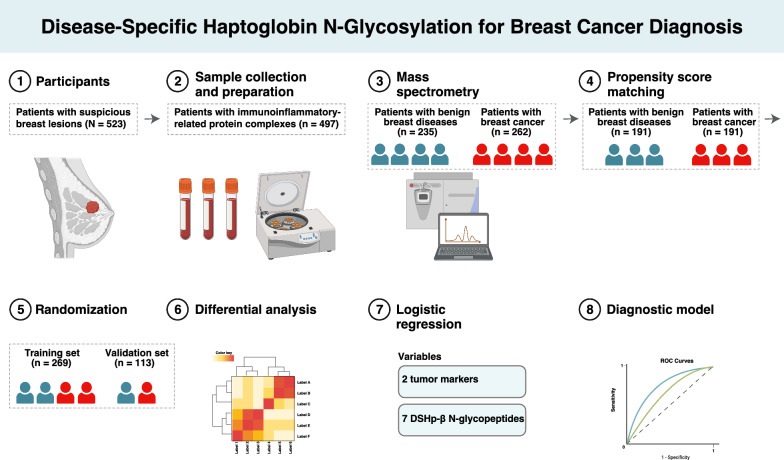
Diagram of the study design

A total of 523 patients were enrolled in the study (Fig. 1). All patients were female, with a median age at surgery of 48.0 (26.0–75.0) years. IIRPC patterns of a, b, c, d, e, f, and g were observed in 54.5%, 36.1%, 5.0%, 2.5%, 0.4%, 1.3%, and 0.2% of the patients, respectively (Table 1). No statistical difference was observed of the IIRPC distribution patterns between patients with benign breast diseases and those with breast cancer (*p* = 0.294). 26 patients were pattern c with no IIRPC, thereby exempted from further mass spectrometric analysis. Of the remaining 497 patients, 235 patients were diagnosed with benign breast diseases and 262 with breast cancer, as confirmed by surgery and pathology. As for pathology, 10 (3.8%), 88 (33.6%), 132 (50.4%), and 32 (12.2%) of the 262 patients with breast cancer were stage 0, I, II, and III, respectively. In addition, 98 (37.4%), 148 (56.5%), and 16 (6.1%) of the malignant cases were low grade (favorable), intermediate grade (moderately favorable), and high grade (unfavorable), respectively. Invasive breast carcinoma of no special type (IBC-NST) represented the most common histologic type, accounting for 72.9% of the malignant cases, while 49.8% and 43.4% of the benign cases were fibroadenoma and adenosis, respectively. As for molecular subtype, 33 (12.6%) of malignant cases were triple-negative, 41 (15.6%) were human epidermal growth factor receptor 2 positive, 89 (34.0%) were of luminal A subtype, and 99 (37.8%) were of luminal B subtype.

**Table 1:** Patterns of serum IIRPCs in patients with benign breast diseases and breast cancer

	a	b	c	d	e	f	g
Benign breast disease n	142	86	16	4	0	3	1
Breast cancer n	143	103	10	9	2	4	0
Total N	285	189	26	13	2	7	1
Percentage %	54.5	36.1	5.0	2.5	0.4	1.3	0.2

After PSM, 191 patients with benign breast diseases and 191 with breast cancer were selected, with the baseline clinical characteristics balanced between groups, which included age, BMI, complete blood count, and blood biochemical markers (Additional file 1: Fig. S3; Table 2). The serum levels of CEA and CA153 were found to be significantly higher in patients with breast cancer compared with those with benign breast diseases, both before and after PSM (*p* < 0.001), while no significant difference was observed for CA125 (*p* = 0.070 and 0.633, respectively, before and after PSM).

### Correlations between DSHp-β N-glycopeptides and tumor markers

In total, 95 site-specific N-glycopeptides were identified by mass spectrometric analysis. Among them, 55 glycoforms were identified at N207/N211, 19 at N241, and 21 at N184 (Additional file 1: Table S1). The correlations of the DSHp-β glycopeptides and tumor markers were illustrated in Additional file 1: Fig. S4. For all cases, numerous significant correlations between DSHp-β glycopeptides were observed. For example, GF2 at N207/N211 positively correlated with G2S2 at N207/N211, as well as G0-N, G-N, G1, G-NS, G2, G2S, GN2F2, G3NS at N241 (R > 0.95). G2S2 at N207/N211 also positively correlated with G0-N, G-N, G1, G-NS, G2, G2S, GN2F2, G2S2, and G3NS at N241 (R > 0.95). What's more, G4NS showed a positive correlation with GS-N &G-NS at N207/N211 (R = 0.97).

**Table 2:** Baseline clinical characteristics of patients

Characteristic	Unmatched			Propensity score matched		
	Benign	Malignant	P-value	Benign	Malignant	P-value
	(N = 235)	(N = 262)		(n = 191)	(n = 191)	
*Age (years)*						
Mean (SD)	44.6 (11.1)	53.0 (11.8)	< 0.001	47.2 (10.4)	49.7 (11.3)	0.055
Median [Min, Max]	43.0 [26.0, 74.0]	52.5 [29.0, 75.0]		46.0 [26.0, 74.0]	48.0 [29.0, 75.0]	
*BMI (kg/m2)*						
< = 24	159 (67.7%)	157 (59.9%)	0.090	120 (62.8%)	123 (64.4%)	0.832
> 24	76 (32.3%)	105 (40.1%)		71 (37.2%)	68 (35.6%)	
*CEA (ng/mL)*						
Mean (SD)	1.4 (0.7)	2.3 (1.8)	< 0.001	1.5 (0.7)	2.1 (1.6)	< 0.001
Median [Min, Max]	1.3 [0, 3.9]	1.7 [0, 17.2]		1.3 [0.4, 3.9]	1.6 [0, 9.1.0]	
*CA125 (U/mL)*						
Mean (SD)	16.6 (13.1)	18.7 (49.3)	0.070	16.0 (13.4)	20.4 (55.8)	0.633
Median [Min, Max]	13.1 [4.7, 113.0]	11.7 [2.3, 762.0]		12.4 [4.7, 113.0]	12.2 [2.3, 762.0]	
*CA153 (U/mL)*						
Mean (SD)	9.4 (5.0)	13.3 (20.0)	< 0.001	9.4 (4.8)	13.6 (23.0)	< 0.001
Median [Min, Max]	7.8 [0, 30.4]	10.3 [3.1, 320.0]		7.8 [2.8, 30.4]	10.7 [3.10, 320.0]	
*ALT (U/L)*						
Mean (SD)	17.9 (16.1)	17.6 (14.8)	0.430	19.0 (17.2)	17.0 (14.8)	0.349
Median [Min, Max]	13.0 [0, 153.0]	14.0 [0, 131.0]		14.0 [6.0, 153.0]	13.0 [0, 131.0]	
*AST (U/L)*						
Mean (SD)	18.6 (7.6)	19.1 (9.0)	0.625	18.9 (7.9)	18.7 (8.1)	0.464
Median [Min, Max]	17.0 [9.0, 65.0]	17.0 [9.0, 93.0]		17.0 [9.0, 65.0]	17.0 [9.0, 73.0]	
*ChE (kU/L)*						
Mean (SD)	8.8 (1.6)	8.9 (1.6)	0.203	8.9 (1.5)	8.7 (1.5)	0.325
Median [Min, Max]	8.6 [4.8, 13.6]	8.9 [4.3, 13.1]		8.7 [5.8, 13.6]	8.6 [4.9, 13.1]	
*TC (mmol/L)*						
Mean (SD)	4.9 (0.8)	5.1 (1.0)	0.023	5.0 (0.8)	5.1 (1.0)	0.633
Median [Min, Max]	4.9 [1.7, 8.4]	5.0 [2.7, 8.8]		4.9 [1.7, 8.4]	5.0 [2.7, 8.8]	
*TG (mmol/L)*						
Mean (SD)	1.1 (0.8)	1.4 (1.0)	< 0.001	1.2 (0.8)	1.3 (0.9)	0.884
Median [Min, Max]	0.9 [0.3, 7.4]	1.1 [0.3, 7.4]		1.0 [0.3, 7.4]	1.0 [0.3, 6.0]	
*HDLC (mmol/L)*						
Mean (SD)	1.5 (0.3)	1.4 (0.3)	0.228	1.4 (0.3)	1.4 (0.3)	0.857
Median [Min, Max]	1.4 [0.5, 2.5]	1.4 [0.8, 2.3]		1.4 [0.5, 2.5]	1.4 [0.8, 2.2]	
*LDLC (mmol/L)*						
Mean (SD)	3.0 (0.7)	3.2 (0.9)	0.053	3.1 (0.7)	3.1 (0.9)	0.986
Median [Min, Max]	3.0 [0.7, 6.6]	3.1 [1.1, 6.9]		3.1 [0.7, 6.6]	3.0 [1.1, 6.9]	
*ApoA1 (g/L)*						
Mean (SD)	1.6 (0.3)	1.6 (0.2)	0.515	1.6 (0.3)	1.6 (0.2)	0.621
Median [Min, Max]	1.5 [0.8, 3.2]	1.5 [1.1, 2.5]		1.5 [0.8, 3.2]	1.5 [1.1, 2.5]	
*ApoB (g/L*)						
Mean (SD)	0.9 (0.2)	1.0 (0.2)	0.002	0.9 (0.2)	0.9 (0.3)	0.805
Median [Min, Max]	0.9 [0.4, 1.7]	0.9 [0.4, 2.6]		0.9 [0.4, 1.7]	0.9 [0.4, 2.6]	
*Lpa (mg/L)*						
Mean (SD)	138.0 (170.0)	163.0 (176.0)	0.004	143.0 (171.0)	146.0 (151.0)	0.281
Median [Min, Max]	74.0 [21.0, 890.0]	97.5 [19.0, 968.0]		82.0 [21.0, 890.0]	87.0 [19.0, 968.0]	
*FFA (μmol/L)*						
Mean (SD)	604.0 (230.0)	638.0 (252.0)	0.132	613.0 (225.0)	627.0 (246.0)	0.663
Median [Min, Max]	597.0 [103.0, 1370.0]	620.0 [103.0, 1490.0]		604.0 [103.0, 1290.0]	604.0 [103.0, 1310.0]	
*hsCRP (mg/L)*						
Mean (SD)	1.1 (1.5)	1.24 (1.63)	0.166	1.1 (1.6)	1.1 (1.4)	0.856
Median [Min, Max]	0.6 [0, 12.5]	0.7 [0, 13.8]		0.6 [0, 12.5]	0.6 [0, 8.4]	
*Glu (mmol/L)*						
Mean (SD)	5.3 (0.8)	5.5 (1.0)	0.006	5.4 (0.8)	5.5 (1.0)	0.344
Median [Min, Max]	5.2 [4.0, 9.5]	5.3 [4.0, 12.6]		5.2 [4.0, 9.5]	5.3 [4.0, 12.6]	
*WBC (10*^*9*^*/L)*						
Mean (SD)	5.8 (1.6)	5.7 (1.4)	0.971	5.8 (1.5)	5.6 (1.4)	0.197
Median [Min, Max]	5.5 [3.0, 11.5]	5.5 [2.7, 11.5]		5.6 [3.0, 11.1]	5.5 [2.7, 11.1]	
*LY (10*^*9*^*/L)*						
Mean (SD)	1.6 (0.4)	1.6 (0.5)	0.480	1.6 (0.5)	1.6 (0.5)	0.145
Median [Min, Max]	1.5 [0.6, 3.0]	1.5 [0.7, 4.9]		1.5 [0.6, 3.0]	1.5 [0.7, 4.9]	
*MONO (10*^*9*^*/L)*						
Mean (SD)	0.3 (0.1)	0.3 (0.1)	0.060	0.3 (0.1)	0.3 (0.1)	0.910
Median [Min, Max]	0.3 [0.1, 0.8]	0.3 [0.1, 1.0]		0.3 [0.1, 0.8]	0.3 [0.1, 1.0]	
*NEUT (10*^*9*^*/L)*						
Mean (SD)	3.8 (1.3)	3.7 (1.3)	0.896	3.8 (1.3)	3.7 (1.3)	0.220
Median [Min, Max]	3.6 [1.5, 9.2]	3.5 [1.2, 8.9]		3.6 [1.5, 8.9]	3.5 [1.2, 8.9]	
*PLT (10*^*9*^*/L)*						
Mean (SD)	253.0 (57.6)	242.0 (51.7)	0.023	249.0 (57.6)	243.0 (52.5)	0.360
Median [Min, Max]	247.0 [128.0, 441.0]	239.0 [126.0, 434.0]		243.0 [128.0, 441.0]	241.0 [126.0, 434.0]	

### Differences of DSHp-β N-glycosylation between breast cancer and benign breast diseases

**Fig. 2:**
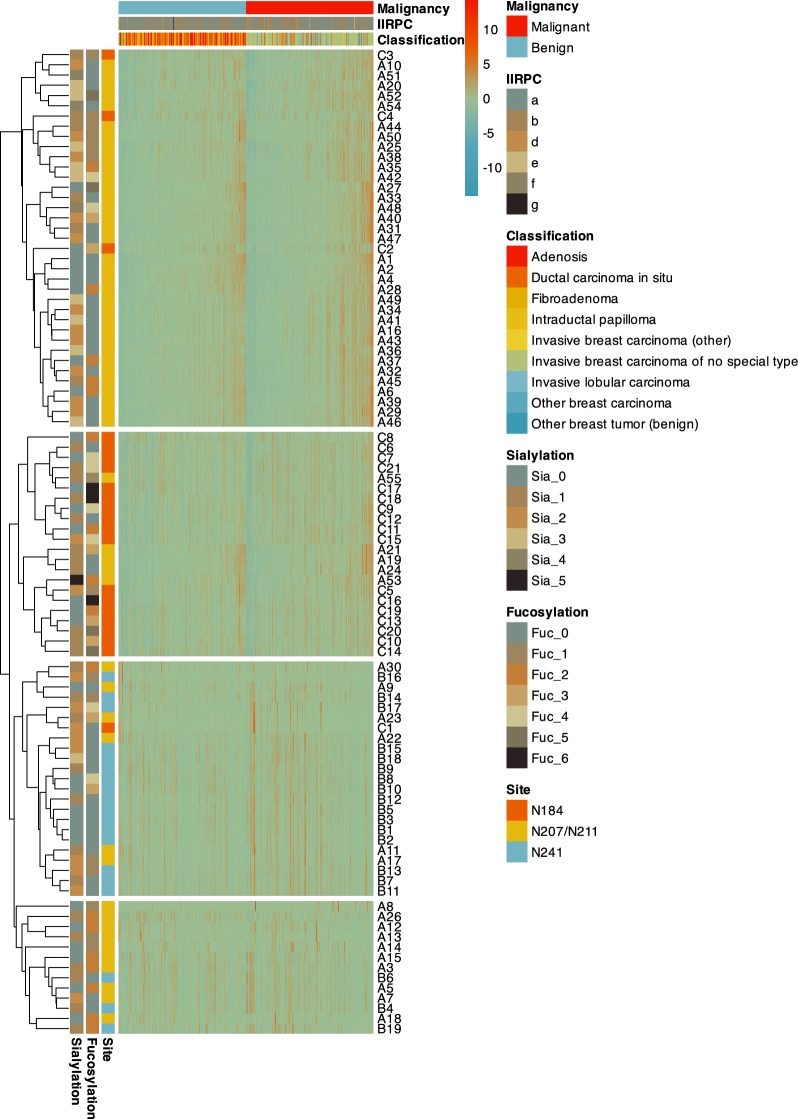
Heatmap of the DSHp-β N-glycopeptides with hierarchical clustering of the rows. Distributions of malignancy, IIRPC patterns, and histologic classifications are demonstrated for each column. Glycosylation sites, fucosylation and sialylation degrees are demonstrated for each row

**Fig. 3:**
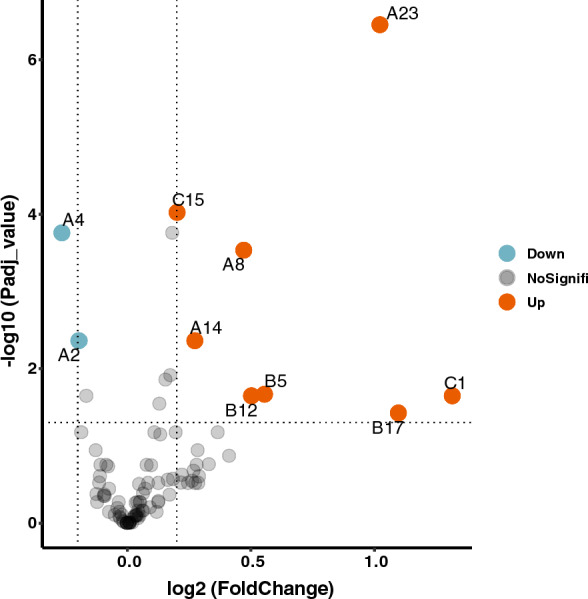
Volcano plot that shows statistical significance (Bonferroni correction p-value) versus magnitude of change (fold change) of DSHp-β N-glycopeptides between benign breast diseases and breast cancer (malignant *vs.* benign). Statistically significant changes are colored

**Fig. 4:**
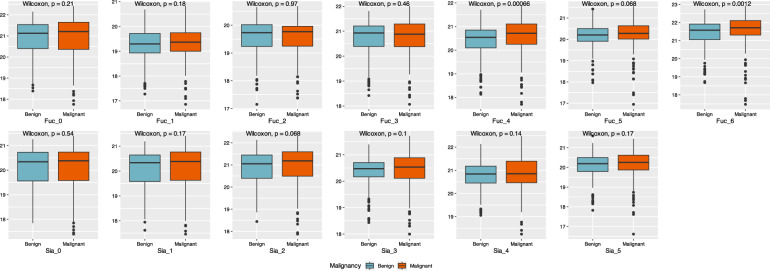
Comparisons of fucosylation and sialylation degrees of DSHp-β N-glycopeptides between benign breast diseases and breast cancer

Numerous significant differences in DSHp-β N-glycosylation between benign breast diseases and breast cancer were observed (Additional file 1: Table S1, Fig. 2). Specifically, significant increases were observed in 8 glycoforms (G2N3F, GN2F5, G2N4F3S at N207/N211, G2, G3NS, G2NF3S2 at N241, and G2S2, G4N3F4S2 at N184) in patients with breast cancer compared with those with benign breast diseases, while significant decreases were observed in 2 glycoforms (G2N2 and GN3 at N207/N211, Fig. 3). The degrees of DSHp-β N-fucosylation and sialylation were measured from the log2-transformed intensities of N-glycopeptides. As a result, the relative intensities of N-tetrafucosyl and hexafucosyl DSHp-β were significantly higher in the malignant group than the benign group (*p* < 0.001, *p* = 0.001, respectively, for tetrafucosyl and hexafucosyl). However, no significant difference in other degrees of fucosylation or sialylation of DSHp-β was found between the two groups (Fig. 4).

### Construction and validation of a new model and nomogram for breast cancer diagnosis

In order to construct the breast cancer diagnostic model, the PSM cohort of 382 patients were randomly divided into the training set (n = 269) and the validation set (n = 113). First, multivariate logistic regression analyses were performed on the training set to study associations between malignancy and clinical characteristics, which included age, BMI, IIRPC patterns, tumor markers, intensities of DSHp-β N-glycosylation, complete blood count, and blood biochemical markers. As a result, 9 variables with no signs of multicollinearity (VIF < 5 for each variable [24]) were selected for model construction (Table 3). In detail, the serum levels of 4 glycoforms (GN2F2 at N184, G-N&G2S2, G2&G3NFS and G2N3F at N207/N211), and tumor markers CEA and CA153 were increased in breast cancer, while the serum levels of 3 glycoforms (G6N3F6, GN2FS at N184, GN3 at N207/N211) were decreased in breast cancer, when compared to benign breast diseases. For the training set, the AUC of the new model to distinguish breast cancer from benign breast diseases was 0.80 (95% confidence interval, CI: 0.75–0.86), with a specificity of 87% and a sensitivity of 62%.

**Table 3:** Selected variables from logistic regression analysis for model construction

Variable	Coef.	OR (95% CI)	P-value	VIF
C11	1.56	4.77 (2.26–10.04)	< 0.001	4.66
A29	1.41	4.11 (2.03–8.34)	< 0.001	4.73
A44	0.61	1.84 (1.24–2.74)	0.003	2.73
A8	0.40	1.50 (1.18–1.90)	0.001	1.10
CEA	0.34	1.40 (1.08–1.82)	0.011	1.04
CA153	0.08	1.09 (1.03–1.14)	0.001	1.06
C16	− 0.86	0.42 (0.24–0.76)	0.004	4.60
C3	− 0.90	0.41 (0.21–0.80)	0.009	3.50
A4	− 2.20	0.11 (0.05–0.25)	< 0.001	4.12

**Fig. 5:**
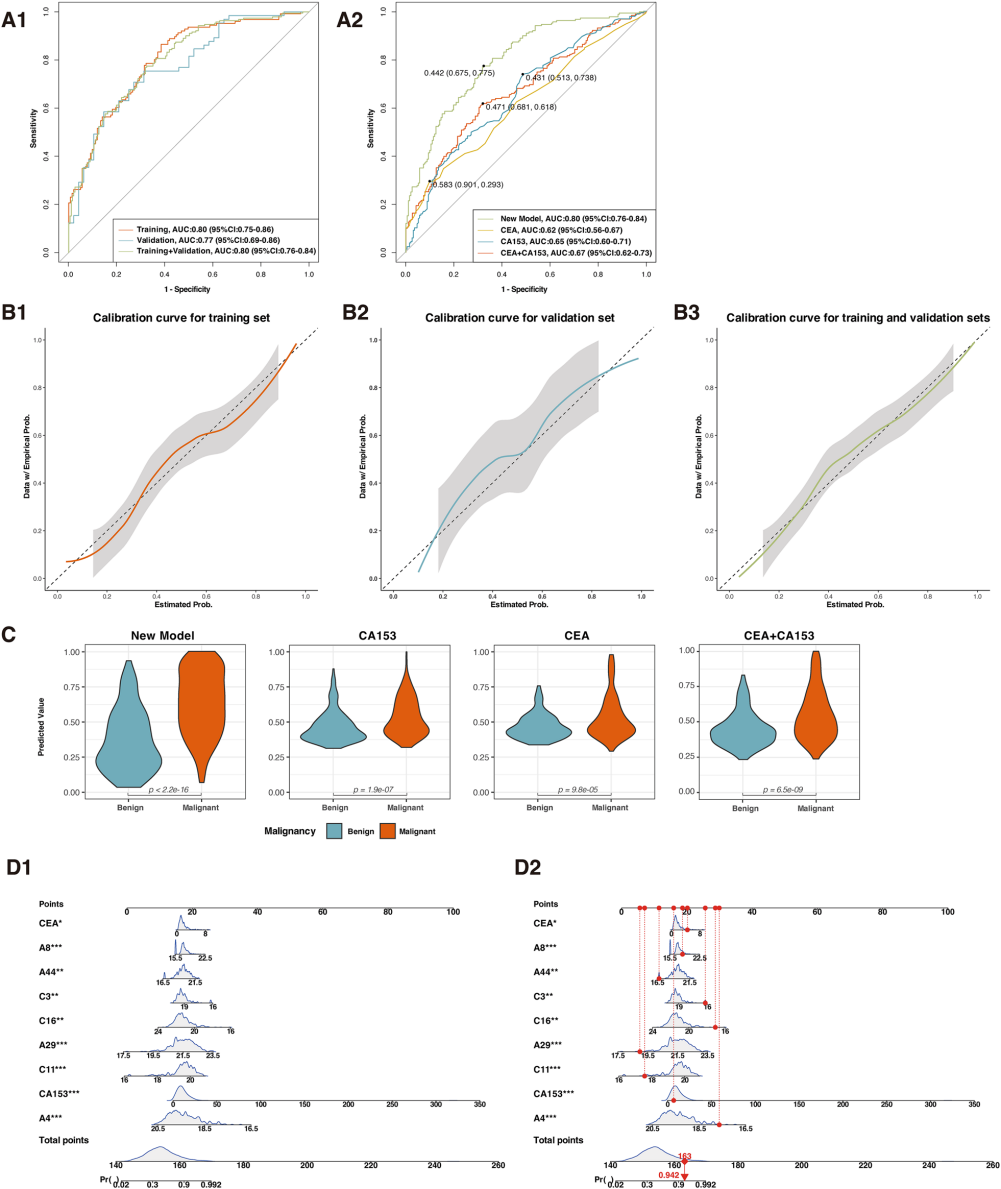
Construction and validation of a new model and nomogram for breast cancer diagnosis. **A1** ROC curves of the new model for predicting breast cancer in the training set, validation set, training and validation sets. **A2** ROC curves of the new model, CA153, CEA, CEA and CA153 for predicting breast cancer in training and validation sets. **B1–3** Calibration curves of the new model for the training set, validation set, training and validation sets. **C** Violin plots that show distributions of predicted values for the new model, CA153, CEA, CEA and CA153. **D1** The nomogram and **D2** its example of the model. The overall probability is calculated by taking the sum of the risk points. For each parameter, its risk point can be determined by drawing a vertical line straight up from the parameter's value to the “Points” axis. In order to determine the probability of breast cancer, a vertical line is drawn intersecting the “Total points” with the “Pr()” line

The diagnostic performance of the new model was subsequently evaluated (Fig. 5A1). For the validation set, the AUC of the new model was 0.77 (95% CI:0.69–0.86, specificity: 75%, sensitivity: 69%). And for training and validation sets, the AUC of the new model was 0.80 (95% CI:0.76–0.84, specificity: 77%, sensitivity: 68%). In general, the calibration curves fitted well with the ideal model for the training set, the validation set, and training and validation sets (Fig. 5B1–3).

To compare the diagnostic performance of the new model and tumor markers CEA and CA153, we draw ROC curves for the new model, CEA and CA153 (Fig. 5A2). With a cutoff value of 2.4 ng/mL, the AUC of CEA was 0.62 (95% CI: 0.56–0.67, specificity: 29%, sensitivity: 90%). With a cutoff value of 7.9 U/mL, the AUC of CA153 was 0.65 (95% CI: 0.60–0.71, specificity: 74%, sensitivity: 51%). For the combination of CEA and CA153, the AUC yielded 0.67 (95% CI: 0.62–0.73, specificity: 60%, sensitivity: 68%). The predicted values of the four models were significantly higher in patients with breast cancer than those with benign diseases (*p* < 0.001, Fig. 5C).

Finally, in order to assess the probability of malignancy, a nomogram was constructed based on the new model. 7 DSHp-β N-glycopeptides (GN2F2, G6N3F6, GN2FS at N184, G-N&G2S2, G2&G3NFS, G2N3F, and GN3 at N207/N211), CEA, and CA153 were used as parameters (Fig. 5D).

## Discussion

To distinguish breast cancer from benign breast diseases at the early stage is of paramount importance for patients with suspicious breast lesions. For those with breast cancer, diagnosis at an earlier stage resulted in reduced mortality and cost from the disease. According to data from the SEER 22 registries, the relative 5-year survival rates for patients with localized and metastatic breast cancer at diagnosis from 2000 to 2019 were 98.9% and 29.2%, respectively [25]. Claim data of 8360 women with breast cancer showed that advanced- versus early-stage breast cancer at diagnosis was associated with significantly increased costs [5]. For patients with benign breast diseases, difficulties in ruling out the cancer diagnosis often result in invasive approaches, including biopsy and surgery. Though defined as the gold standard for diagnosis, such approaches could cause infectious, bleeding and thromboembolic complications at varying incidence [26, 27]. Despite improvements in surgical techniques, according to a study on 226,899 patients from the American College of Surgeons NSQIP database who underwent breast surgery from 2005 to 2017, the overall rates of acute complication were 2.25–3.2% for breast conserving therapy, and 5.68–13.04% for mastectomy, respectively [27]. In addition, literature reported a 10–50% cumulative incidence of lymphedema 2 years after breast cancer surgery [28, 29].

In this scenario, it is pivotal to explore efficient approaches for differential diagnosis of breast cancer. In this study, we demonstrate for the first time that quantitative analysis of serum DSHp-β N-glycosylation can aid current tumor markers in differentiating breast cancer from benign breast diseases. With the addition of preoperative serum levels of 7 glycopeptides to CEA and CA153, our new model yielded an AUC of 0.80. In comparison, the calculated AUCs of the same cohort were 0.62, 0.65, and 0.67, respectively, for CEA, CA153, as well as their combination. We distinguished stage 0-III breast cancer from suspicious breast lesions, instead of normal controls, which could better address the clinical need. The performance of the new model for disease monitoring in patients with breast cancer remains to be explored.

IIRPC is a group of immunoinflammatory-related and non-covalently linked proteins, reported to be associated with the development and progression of cancer [23, 30]. Major components of IIRPCs are complements, immunoglobulins, and haptoglobin. In consistent with previous studies, there were 7 patterns of the IIRPCs observed [23, 31]. No significant difference in the IIRPC patterns between benign and malignant cases was found. We didn't quantify IIRPCs, though they were potentially related with treatment response in breast cancer [31]. It's well established that protein glycosylation, as one of the post-translational modifications, is highly sensitive and closely related to tumorigenesis and disease evolution [32]. In particular, abnormal fucosylation and sialylation of Hp could contribute to tumorigenesis, cancer progression, and metastasis [15–19]. In our study, DSHp-β N-glycopeptides were found to be closely correlated, which is understandable in view of critical enzymes responsible for their modification [33]. Specifically, the upregulation of N-tetrafucosyl and hexafucosyl DSHp-β indicates an activation of fucosyltransferase 8 (FUT8) in tumorigenesis [34]. This was in accordance with the report that FUT8 expression was elevated in breast cancer through transcription factor activator protein 2γ regulation [35]. The dysregulated N-glycosylation profiles of DSHp-β suggest an underlying inflammatory response in breast cancer that awaits further investigation. Because of relative large number of the glycopeptides and their multicollinearity, we used logistic regression instead of other machine learning methods for model construction. And probably due to the multicollinearity of DSHp-β N-glycopeptides, not all differentially expressed genes were selected for model construction.

Our study found significant differences in serum levels of CEA and CA153 between breast cancer and benign breast diseases. However, CA125 was not significantly increased in breast cancer compared to benign breast diseases, neither before nor after PSM. CA125 is a biomarker for ovarian cancer, usually associated with breast cancer in the metastatic settings [36, 37]. Furthermore, we evaluated the diagnostic performance of CEA and CA153 in breast cancer. CA153 provided a higher specificity (74%) than sensitivity (51%), while CEA provided a high sensitivity (90%) but a low specificity (29%). CA153 can be elevated in benign breast diseases, therefore is not sensitive enough for early detection of breast cancer [38]. On the contrary, CEA is very sensitive but can be elevated in many other malignancies (e.g., colon cancer, lung cancer, pancreatic cancer, thyroid cancer, etc.). Hence, CEA and CA153 could complement each other for breast cancer diagnosis. When combined, their sensitivity and specificity yielded 68% and 60%, respectively. In our study, the selected cutoff value of CA153 (7.9 U/mL) was lower than the normal limit (25 U/mL). This is probably because we didn't include patients with metastasis. CA153 levels were observed to increase with the tumor stage [39]. In fact, CA153 levels in patients with localized breast cancer largely overlapped those in healthy women or patients with benign breast diseases [39–41].

The study has some strengths and limitations. First, the study obtained detailed clinical information of the participants, and used PSM and randomization to effectively control for confounding. The enrolled participants largely resembled those of the clinical reality, therefore the results of the study could be of practical use. Second, the ultra-resolution MS applied in our study required only a small volume of blood, used an economical and environmental-friendly enrichment method, and proved to be quantitatively reproducible [18, 19], indicating high potential clinical applications. Of note, the single-center retrospective design of the study was prone to selection bias. Although the model and nomogram performed well through internal validation, prospective and external validation on an expanded population is expected to provide more convincing evidence in future. Furthermore, the role of aberrant glycosylation in relation to drug response and prognosis of breast cancer is expected to be investigated with follow-ups of the patients.

## Conclusions

The N-glycosylation profile of serum DSHp-β was largely altered in patients with breast cancer. Based on the preoperative serum levels of 7 DSHp-β N-glycopeptides, CEA, and CA153, we developed a promising model and nomogram to differentiate between breast cancer and benign breast diseases. The diagnostic performance of the new model and nomogram was better than traditional tumor markers. Overall, advances in the understanding of DSHp-β N-glycosylation could offer insights into breast tumorigenesis and guide clinical practice.

### Supplementary Information

**Additional file 1: Figure S1.** Aggregation plots of missing values of clinical variables. The first plot shows the proportion of missing values in each variable. The second plot shows patterns of missing values. The frequencies of the corresponding combinations are demonstrated to the right. The blue bars represent missing values, while the orange bars represent observed values. **Figure S2.** Strip plots of observed and imputed data of clinicopathological variables. The strip plots display the distribution of imputed values (orange points) over observed values (blue points) in a combined way. In total, 5 multiple imputed data sets were created. Column 1 represents the original data set, while column 2-6 represent the 5 imputed data sets. The second imputed data set (column 3) was used. Most of its imputations were in a plausible range, and properly accounted for the distribution of the missing data. **Figure S3.** Histogram plots displaying propensity score distributions for the malignant and benign groups before and after propensity score matching (caliper = 0.333). **Figure S4.** Heatmap of the correlations of DSHp-β N-glycopeptides and tumor markers. The numbers in grid show the Spearman correlation coefficients. Blank indicates a Bonferroni correction p-value of ≥ 0.05. **Table S1.** Identified N-glycopeptides of DSHp-β, their potential structures, and intensities between benign breast diseases and breast cancer.

## Data Availability

Data are available by contacting the corresponding authors.
