# Supplementary material for: Development and validation of a model and nomogram for breast cancer diagnosis based on quantitative analysis of serum disease-specific haptoglobin N-glycosylation
Source: J Transl Med. 2024 Apr 4;22:331. doi: 10.1186/s12967-024-05039-4 (PMC10993522; doi:10.1186/s12967-024-05039-4)
Supplement: Supplementary file 1 — Additional file 1: Figure S1. Aggregation plots of missing values of clinical variables. The first plot shows the proportion of missing values in each variable. The second plot shows patterns of missing values. The frequencies of the corresponding combinations are demonstrated to the right. The blue bars represent missing values, while the orange bars represent observed values. Figure S2. Strip plots of observed and imputed data of clinicopathological variables. The strip plots display the distribution of imputed values (orange points) over observed values (blue points) in a combined way. In total, 5 multiple imputed data sets were created. Column 1 represents the original data set, while column 2-6 represent the 5 imputed data sets. The second imputed data set (column 3) was used. Most of its imputations were in a plausible range, and properly accounted for the distribution of the missing data. Figure S3. Histogram plots displaying propensity score distributions for the malignant and benign groups before and after propensity score matching (caliper = 0.333). Figure S4. Heatmap of the correlations of DSHp-β N-glycopeptides and tumor markers. The numbers in grid show the Spearman correlation coefficients. Blank indicates a Bonferroni correction p-value of ≥ 0.05. Table S1. Identified N-glycopeptides of DSHp-β, their potential structures, and intensities between benign breast diseases and breast cancer. [file 12967_2024_5039_MOESM1_ESM.docx]

**Additional Data**

 Figure S1. Aggregation plots of missing values of clinical variables. The first plot shows the proportion of missing values in each variable. The second plot shows patterns of missing values. The frequencies of the corresponding combinations are demonstrated to the right. The blue bars represent missing values, while the orange bars represent observed values.

Figure S2. Strip plots of observed and imputed data of clinicopathological variables. The strip plots display the distribution of imputed values (orange points) over observed values (blue points) in a combined way. In total, 5 multiple imputed data sets were created. Column 1 represents the original data set, while column 2-6 represent the 5 imputed data sets. The second imputed data set (column 3) was used. Most of its imputations were in a plausible range, and properly accounted for the distribution of the missing data.

Figure S3. Histogram plots displaying propensity score distributions for the malignant and benign groups before and after propensity score matching (caliper = 0.333).

Figure S4. Heatmap of the correlations of DSHp-β N-glycopeptides and tumor markers. The numbers in grid show the Spearman correlation coefficients. Blank indicates a Bonferroni correction p-value of ≥ 0.05.

Table S1. Identified N-glycopeptides of DSHp-β, their potential structures, and intensities between benign breast diseases and breast cancer.

| **Ref** | **Glycoform** | **Experimental mass** | **Theoretical mass** | **Error (ppm)** | **Structure** | **Log2 intensity Mean (SD)** | | **log2FoldChange** | **P-value** | **Bonferroni correction p-value** |
| --- | --- | --- | --- | --- | --- | --- | --- | --- | --- | --- |
|  |  |  |  |  |  | **Benign** | **Malignant** |  |  |  |
| **NLFLN207HSEN211ATAK** | | | | | |  | | | |  |
| A1 | G2N | 3284.4 | 3284.4 | -3.8 | (Hex)2 (HexNAc)3 + (Man)3(GlcNAc)2 | 21.6 (0.7) | 21.5 (0.8) | -0.166 | 0.003 | 0.023 |
| A2 | G2N2 | 3487.5 | 3487.5 | -4.6 | (Hex)2 (HexNAc)4 + (Man)3(GlcNAc)2 | 19.7 (0.5) | 19.6 (0.7) | -0.195 | <0.001 | 0.004 |
| A3 | GF2S | 3502.5 | 3502.5 | 8.7 | (Hex)1 (HexNAc)2 (Deoxyhexose)2 (NeuAc)1 + (Man)3(GlcNAc)2 | 17.3 (1.1) | 17.3 (1.2) | 0.049 | 0.629 | 0.796 |
| A4 | GN3 | 3528.5 | 3528.5 | -4.6 | (Hex)1 (HexNAc)5 + (Man)3(GlcNAc)2 | 19.5 (0.5) | 19.3 (0.7) | -0.264 | <0.001 | <0.001 |
| A5 | GF2 | 3543.5 | 3543.5 | -7.7 | (Hex)1 (HexNAc)2 (Deoxyhexose)2 + (Man)3(GlcNAc)2 | 19.2 (1.7) | 19.1 (1.9) | 0.217 | 0.120 | 0.299 |
| A6 | G2NF2 | 3576.5 | 3576.5 | 5.1 | (Hex)2 (HexNAc)3 (Deoxyhexose)2 + (Man)3(GlcNAc)2 | 21.6 (0.8) | 21.6 (0.9) | -0.114 | 0.123 | 0.299 |
| A7 | G2S2 | 3663.5 | 3663.5 | 2.0 | (Hex)2 (HexNAc)2 (NeuAc)2 + (Man)3(GlcNAc)2 | 19.7 (1.7) | 19.8 (2.0) | 0.28 | 0.047 | 0.177 |
| A8 | G2N3F | 3834.6 | 3834.6 | -7.0 | (Hex)2 (HexNAc)5 (Deoxyhexose)1 + (Man)3(GlcNAc)2 | 17.2 (0.9) | 17.5 (1.2) | 0.471 | <0.001 | <0.001 |
| A9 | G3N3 | 3852.6 | 3852.6 | -5.8 | (Hex)3 (HexNAc)5 + (Man)3(GlcNAc)2 | 19.0 (1.1) | 19.0 (1.3) | 0.091 | 0.405 | 0.620 |
| A10 | G2NS2 | 3866.6 | 3866.6 | 9.8 | (Hex)2 (HexNAc)3 (NeuAc)2 + (Man)3(GlcNAc)2 | 21.3 (0.6) | 21.3 (0.8) | -0.078 | 0.058 | 0.185 |
| A11 | G4NS | 3897.5 | 3897.6 | -8.4 | (Hex)4 (HexNAc)3 (NeuAc)1 + (Man)3(GlcNAc)2 | 21.2 (1.9) | 21.3 (2.2) | 0.29 | 0.089 | 0.248 |
| A12 | G5F2 | 4021.6 | 4021.6 | -1.9 | (Hex)6 (HexNAc)2 (Deoxyhexose)2 + (Man)3(GlcNAc)2 | 20.8 (2.1) | 20.7 (2.3) | 0.019 | 0.907 | 0.981 |
| A13 | G4NFS | 4043.6 | 4043.6 | -5.7 | (Hex)4 (HexNAc)3 (Deoxyhexose)1 (NeuAc)1 + (Man)3(GlcNAc)2 | 18.5 (1.3) | 18.3 (1.3) | -0.123 | 0.327 | 0.531 |
| A14 | GN2F5 | 4055.7 | 4055.7 | 5.9 | (Hex)1 (HexNAc)4 (Deoxyhexose)5 + (Man)3(GlcNAc)2 | 17.6 (0.9) | 17.9 (0.9) | 0.273 | <0.001 | 0.004 |
| A15 | G3N3F2 | 4144.8 | 4144.7 | 8.3 | (Hex)3 (HexNAc)5 (Deoxyhexose)2 + (Man)3(GlcNAc)2 | 19.2 (1.0) | 19.2 (1.0) | 0.125 | 0.132 | 0.301 |
| A16 | G3N2S2 | 4231.7 | 4231.7 | -6.2 | (Hex)3 (HexNAc)4 (NeuAc)2 + (Man)3(GlcNAc)2 | 20.1 (0.6) | 20.2 (0.9) | -0.002 | 0.981 | 0.990 |
| A17 | G2N2FS2 | 4377.8 | 4377.8 | 0.1 | (Hex)3 (HexNAc)4 (Deoxyhexose)1 (NeuAc)2 + (Man)3(GlcNAc)2 | 20.5 (1.6) | 20.8 (1.8) | 0.268 | 0.069 | 0.211 |
| A18 | G7NF2 | 4386.8 | 4386.8 | 5.1 | (Hex)7 (HexNAc)3 (Deoxyhexose)2 + (Man)3(GlcNAc)2 | 19.1 (1.4) | 18.9 (1.4) | -0.075 | 0.502 | 0.711 |
| A19 | G-N&GS | 4467.8 | 4467.8 | -2.0 | (Hex)5 (HexNAc)5 (NeuAc)1 + (Man)3(GlcNAc)2 | 22.7 (0.7) | 22.8 (0.9) | 0.006 | 0.897 | 0.981 |
| A20 | GN2S3 | 4522.8 | 4522.8 | -3.8 | (Hex)3 (HexNAc)4 (NeuAc)3 + (Man)3(GlcNAc)2 | 19.9 (0.5) | 20.0 (0.7) | -0.035 | 0.336 | 0.531 |
| A21 | G4N2F3S | 4540.8 | 4540.8 | -9.0 | (Hex)4 (HexNAc)4 (Deoxyhexose)3 (NeuAc)1 + (Man)3(GlcNAc)2 | 21.2 (0.7) | 21.2 (0.8) | -0.032 | 0.515 | 0.711 |
| A22 | GS-N&G-NS | 4553.8 | 4553.8 | 0.8 | (Hex)5 (HexNAc)4 (NeuAc)2 + (Man)3(GlcNAc)2 | 19.5 (1.5) | 19.7 (1.7) | 0.125 | 0.327 | 0.531 |
| A23 | G2N4F3S | 4622.9 | 4622.9 | 2.6 | (Hex)2 (HexNAc)6 (Deoxyhexose)3 (NeuAc)1 + (Man)3(GlcNAc)2 | 18.4 (1.4) | 18.7 (1.9) | 1.022 | <0.001 | <0.001 |
| A24 | G-N&G2S | 4629.8 | 4629.9 | -7.0 | (Hex)6 (HexNAc)5 (NeuAc)1 + (Man)3(GlcNAc)2 | 22.8 (0.8) | 22.9 (0.9) | 0.052 | 0.332 | 0.531 |
| A25 | G3N2FS3 | 4668.8 | 4668.9 | -6.9 | (Hex)3 (HexNAc)4 (Deoxyhexose)1 (NeuAc)3 + (Man)3(GlcNAc)2 | 18.8 (0.6) | 18.7 (0.8) | -0.11 | 0.050 | 0.177 |
| A26 | G7NF2S | 4677.8 | 4677.9 | -8.3 | (Hex)7 (HexNAc)3 (Deoxyhexose)2 (NeuAc)1 + (Man)3(GlcNAc)2 | 18.8 (0.9) | 18.7 (0.8) | -0.187 | 0.012 | 0.067 |
| A27 | G5N2F5 | 4703.9 | 4703.9 | -6.0 | (Hex)5 (HexNAc)4 (Deoxyhexose)5 + (Man)3(GlcNAc)2 | 21.4 (0.9) | 21.4 (1.0) | -0.096 | 0.244 | 0.443 |
| A28 | G0F&GNF | 4713.0 | 4713.1 | -6.1 | (Hex)4 (HexNAc)7 (Deoxyhexose)2 + (Man)3(GlcNAc)2 | 19.4 (0.6) | 19.4 (0.7) | -0.129 | 0.026 | 0.114 |
| A29 | G-N&G2S2 | 4758.9 | 4758.9 | -8.3 | (Hex)5 (HexNAc)5 (NeuAc)2 + (Man)3(GlcNAc)2 | 21.5 (0.7) | 21.6 (0.9) | 0.017 | 0.766 | 0.888 |
| A30 | G0F&GFS | 4801.0 | 4801.0 | 5.4 | (Hex)4 (HexNAc)6 (Deoxyhexose)2 (NeuAc)1 + (Man)3(GlcNAc)2 | 17.9 (1.1) | 18.0 (1.1) | 0.01 | 0.919 | 0.981 |
| A31 | G1S&G2 | 4833.0 | 4832.9 | 9.2 | (Hex)6 (HexNAc)6 (NeuAc)1 + (Man)3(GlcNAc)2 | 21.3 (0.7) | 21.4 (0.9) | 0.028 | 0.681 | 0.819 |
| A32 | G2S2&G-N | 4921.0 | 4921.0 | 8.4 | (Hex)6 (HexNAc)5 (NeuAc)2 + (Man)3(GlcNAc)2 | 20.9 (0.8) | 20.9 (1.0) | -0.015 | 0.848 | 0.960 |
| A33 | G2S&G2 | 4995.0 | 4995.0 | -5.1 | (Hex)7 (HexNAc)6 (NeuAc)1 + (Man)3(GlcNAc)2 | 22.9 (1.0) | 23.1 (1.0) | 0.043 | 0.600 | 0.781 |
| A34 | G2S2&G1 | 5124.0 | 5124.0 | -8.0 | (Hex)6 (HexNAc)6 (NeuAc)2 + (Man)3(GlcNAc)2 | 21.1 (0.7) | 21.0 (1.1) | -0.029 | 0.724 | 0.859 |
| A35 | G5N2F2S3 | 5139.0 | 5139.0 | -9.7 | (Hex)5 (HexNAc)4 (Deoxyhexose)2 (NeuAc)3 + (Man)3(GlcNAc)2 | 19.7 (0.8) | 19.7 (0.9) | -0.003 | 0.970 | 0.990 |
| A36 | G2S2&GS | 5212.0 | 5212.1 | -8.7 | (Hex)6 (HexNAc)5 (NeuAc)3 + (Man)3(GlcNAc)2 | 19.3 (0.7) | 19.3 (1.0) | -0.018 | 0.812 | 0.929 |
| A37 | GN2F&G2F | 5240.3 | 5240.3 | 6.2 | (Hex)6 (HexNAc)8 (Deoxyhexose)2 + (Man)3(GlcNAc)2 | 20.8 (0.8) | 20.8 (1.0) | -0.092 | 0.247 | 0.443 |
| A38 | G2S2&GF | 5270.0 | 5270.1 | -8.3 | (Hex)6 (HexNAc)6 (Deoxyhexose)1 (NeuAc)2 + (Man)3(GlcNAc)2 | 19.8 (0.6) | 19.7 (1.0) | -0.033 | 0.675 | 0.819 |
| A39 | G2S2&G2 | 5286.1 | 5286.1 | 9.8 | (Hex)7 (HexNAc)6 (NeuAc)2 + (Man)3(GlcNAc)2 | 23.4 (0.8) | 23.5 (0.9) | 0.008 | 0.917 | 0.981 |
| A40 | G2F2S&G-NFS | 5359.1 | 5359.1 | -7.3 | (Hex)6 (HexNAc)5 (Deoxyhexose)3 (NeuAc)2 + (Man)3(GlcNAc)2 | 21.8 (0.9) | 21.8 (1.2) | -0.051 | 0.585 | 0.781 |
| A41 | G2S2&GS | 5415.2 | 5415.1 | 8.7 | (Hex)6 (HexNAc)6 (NeuAc)3 + (Man)3(GlcNAc)2 | 20.1 (0.7) | 20.2 (0.9) | 0.036 | 0.637 | 0.796 |
| A42 | G5N2F4S3 | 5431.1 | 5431.2 | -6.2 | (Hex)5 (HexNAc)4 (Deoxyhexose)4 (NeuAc)3 + (Man)3(GlcNAc)2 | 20.4 (0.8) | 20.5 (1.0) | -0.004 | 0.963 | 0.990 |
| A43 | GS&G3S | 5489.2 | 5489.2 | 5.9 | (Hex)7 (HexNAc)7 (NeuAc)2 + (Man)3(GlcNAc)2 | 20.2 (0.8) | 20.3 (1.0) | 0.034 | 0.651 | 0.803 |
| A44 | G2&G3NFS | 5506.2 | 5506.2 | 6.4 | (Hex)8 (HexNAc)7 (Deoxyhexose)1 (NeuAc)1 + (Man)3(GlcNAc)2 | 19.6 (0.9) | 19.6 (1.1) | 0.01 | 0.912 | 0.981 |
| A45 | GN2FS&G3F | 5531.3 | 5531.4 | -6.8 | (Hex)6 (HexNAc)8 (Deoxyhexose)2 (NeuAc)1 + (Man)3(GlcNAc)2 | 20.6 (0.8) | 20.5 (1.0) | -0.094 | 0.223 | 0.423 |
| A46 | G2S2&G2S | 5577.1 | 5577.2 | -6.2 | (Hex)7 (HexNAc)6 (NeuAc)3 + (Man)3(GlcNAc)2 | 23.4 (1.2) | 23.5 (1.3) | 0.064 | 0.471 | 0.685 |
| A47 | G2S&G3NS | 5651.2 | 5651.2 | -7.7 | (Hex)8 (HexNAc)7 (NeuAc)2 + (Man)3(GlcNAc)2 | 23.3 (1.1) | 23.4 (1.2) | 0.06 | 0.476 | 0.685 |
| A48 | G5N2F4S4 | 5722.2 | 5722.2 | -7.1 | (Hex)5 (HexNAc)4 (Deoxyhexose)4 (NeuAc)4 + (Man)3(GlcNAc)2 | 21.0 (0.7) | 21.1 (1.0) | 0.037 | 0.598 | 0.781 |
| A49 | GS&G3NS2 | 5780.2 | 5780.3 | -8.4 | (Hex)7 (HexNAc)7 (NeuAc)3 + (Man)3(GlcNAc)2 | 19.9 (0.6) | 20.1 (0.8) | 0.043 | 0.464 | 0.685 |
| A50 | G2S2&G3NF | 5797.2 | 5797.3 | -7.7 | (Hex)8 (HexNAc)7 (Deoxyhexose)1 (NeuAc)2 + (Man)3(GlcNAc)2 | 21.2 (0.9) | 21.3 (1.1) | 0.007 | 0.931 | 0.982 |
| A51 | G2S2&G2S2 | 5868.2 | 5868.3 | -7.2 | (Hex)7 (HexNAc)6 (NeuAc)4 + (Man)3(GlcNAc)2 | 21.6 (0.8) | 21.7 (0.9) | -0.003 | 0.964 | 0.990 |
| A52 | GF2S-N &G2F3S2 | 5942.4 | 5942.3 | 5.5 | (Hex)6 (HexNAc)5 (Deoxyhexose)5 (NeuAc)3 + (Man)3(GlcNAc)2 | 19.8 (0.5) | 19.9 (0.7) | -0.001 | 0.990 | 0.990 |
| A53 | G7N2F2S5 | 6045.4 | 6045.3 | 10.0 | (Hex)7 (HexNAc)4 (Deoxyhexose)2 (NeuAc)5 + (Man)3(GlcNAc)2 | 20.1 (0.6) | 20.1 (0.7) | -0.022 | 0.596 | 0.781 |
| A54 | G2S2&G2NS2 | 6070.4 | 6070.3 | 10.0 | (Hex)7 (HexNAc)7 (NeuAc)4 + (Man)3(GlcNAc)2 | 19.6 (0.5) | 19.8 (0.7) | 0.033 | 0.348 | 0.542 |
| A55 | G2N2&G4N3FS | 6480.6 | 6480.6 | 9.8 | (Hex)9 (HexNAc)11 (Deoxyhexose)1 (NeuAc)1 + (Man)3(GlcNAc)2 | 19.4 (0.7) | 19.6 (0.8) | 0.078 | 0.052 | 0.177 |
| **VVLHPN241YSQVDIGLIK** | | | | | |  | | | |  |
| B1 | G0-N | 2890.4 | 2890.4 | 4.0 | (HexNAc)1 + (Man)3(GlcNAc)2 | 19.5 (2.0) | 19.6 (2.2) | 0.262 | 0.110 | 0.283 |
| B2 | G-N | 3052.5 | 3052.5 | 4.9 | (Hex)1 (HexNAc)1 + (Man)3(GlcNAc)2 | 19.3 (2.0) | 19.3 (2.3) | 0.33 | 0.044 | 0.173 |
| B3 | G1 | 3255.5 | 3255.5 | -4.0 | (Hex)1 (HexNAc)2 + (Man)3(GlcNAc)2 | 18.6 (1.6) | 18.5 (1.9) | 0.171 | 0.229 | 0.426 |
| B4 | G-NS | 3343.6 | 3343.6 | 7.1 | (Hex)1 (HexNAc)1 (NeuAc)1 + (Man)3(GlcNAc)2 | 19.6 (1.9) | 19.7 (2.1) | 0.366 | 0.013 | 0.067 |
| B5 | G2 | 3417.6 | 3417.6 | 7.4 | (Hex)2 (HexNAc)2 + (Man)3(GlcNAc)2 | 20.7 (2.3) | 20.8 (2.7) | 0.556 | 0.002 | 0.021 |
| B6 | GS | 3692.7 | 3692.7 | 4.0 | (Hex)1 (HexNAc)2 (Deoxyhexose)1 (NeuAc)1 + (Man)3(GlcNAc)2 | 17.9 (1.1) | 17.9 (1.2) | 0.164 | 0.104 | 0.275 |
| B7 | G2S | 3708.7 | 3708.7 | -6.4 | (Hex)2 (HexNAc)2 (NeuAc)1 + (Man)3(GlcNAc)2 | 23.4 (2.9) | 23.4 (3.4) | 0.412 | 0.033 | 0.135 |
| B8 | G0NF4 | 3880.8 | 3880.8 | -7.5 | (HexNAc)3 (Deoxyhexose)4 + (Man)3(GlcNAc)2 | 18.5 (1.4) | 18.6 (1.6) | 0.125 | 0.292 | 0.515 |
| B9 | G2NS | 3911.7 | 3911.8 | -8.4 | (Hex)2 (HexNAc)3 (NeuAc)1 + (Man)3(GlcNAc)2 | 18.2 (1.5) | 18.2 (1.6) | 0.042 | 0.732 | 0.859 |
| B10 | GN2F2 | 3953.8 | 3953.8 | 8.7 | (Hex)1 (HexNAc)4 (Deoxyhexose)2 + (Man)3(GlcNAc)2 | 21.8 (2.6) | 21.7 (2.9) | 0.118 | 0.517 | 0.711 |
| B11 | G2S2 | 3999.7 | 3999.8 | -8.5 | (Hex)2 (HexNAc)2 (NeuAc)2 + (Man)3(GlcNAc)2 | 24.5 (3.1) | 24.5 (3.5) | 0.287 | 0.137 | 0.301 |
| B12 | G3NS | 4073.8 | 4073.8 | 5.7 | (Hex)3 (HexNAc)3 (NeuAc)1 + (Man)3(GlcNAc)2 | 20.6 (2.2) | 20.8 (2.5) | 0.503 | 0.003 | 0.023 |
| B13 | GNFS2 | 4186.9 | 4186.9 | 6.0 | (Hex)1 (HexNAc)3 (Deoxyhexose)1 (NeuAc)2 + (Man)3(GlcNAc)2 | 20.3 (1.6) | 20.5 (1.8) | 0.284 | 0.026 | 0.114 |
| B14 | G3NFS | 4219.8 | 4219.9 | -7.1 | (Hex)3 (HexNAc)3 (Deoxyhexose)1 (NeuAc)1 + (Man)3(GlcNAc)2 | 17.0 (1.2) | 17.1 (1.2) | 0.185 | 0.099 | 0.268 |
| B15 | G3NS2 | 4364.9 | 4364.9 | 6.4 | (Hex)3 (HexNAc)3 (NeuAc)2 + (Man)3(GlcNAc)2 | 22.5 (2.7) | 22.6 (3.1) | 0.274 | 0.136 | 0.301 |
| B16 | G3NFS2 | 4510.9 | 4511.0 | -7.6 | (Hex)3 (HexNAc)3 (Deoxyhexose)1 (NeuAc)2 + (Man)3(GlcNAc)2 | 19.2 (1.6) | 19.2 (1.6) | 0.221 | 0.080 | 0.236 |
| B17 | G2NF3S2 | 4641.1 | 4641.0 | 7.5 | (Hex)2 (HexNAc)3 (Deoxyhexose)3 (NeuAc)2 + (Man)3(GlcNAc)2 | 19.5 (1.8) | 20.0 (2.2) | 1.097 | 0.006 | 0.038 |
| B18 | G3NS3 | 4656.0 | 4656.0 | 7.8 | (Hex)3 (HexNAc)3 (NeuAc)3 + (Man)3(GlcNAc)2 | 22.3 (2.3) | 22.4 (2.5) | 0.246 | 0.140 | 0.301 |
| B19 | G4N2F2S | 4731.1 | 4731.1 | 6.3 | (Hex)4 (HexNAc)4 (Deoxyhexose)2 (NeuAc)1 + (Man)3(GlcNAc)2 | 18.6 (0.8) | 18.8 (0.9) | 0.196 | 0.012 | 0.067 |
| **MVSHHN184LTTGATLINEQWLL TTAK** | | | | | |  | | | |  |
| C1 | G2S2 | 4609.0 | 4609.1 | -5.7 | (Hex)2 (HexNAc)2 (NeuAc)1 + (Man)3(GlcNAc)2 | 18.9 (2.5) | 19.4 (2.9) | 1.315 | 0.003 | 0.023 |
| C2 | G3N2F3 | 5308.3 | 5308.4 | -8.3 | (Hex)3 (HexNAc)4 (Deoxyhexose)3 + (Man)3(GlcNAc)2 | 20.3 (0.8) | 20.1 (1.2) | -0.126 | 0.216 | 0.418 |
| C3 | GN2FS | 6058.9 | 6058.8 | 8.7 | (HexNAc)3 (Deoxyhexose)3 + (Man)3(GlcNAc)2 | 19.1 (0.6) | 19.1 (0.8) | -0.086 | 0.050 | 0.177 |
| C4 | GN3FS | 6109.8 | 6109.7 | 9.6 | (Hex)3 (HexNAc)2 (Deoxyhexose)1 (NeuAc)2 + (Man)3(GlcNAc)2 | 19.7 (0.7) | 19.6 (0.8) | -0.111 | 0.086 | 0.246 |
| C5 | G3FS2 | 6160.9 | 6160.8 | 8.9 | (Hex)1 (HexNAc)6 (NeuAc)1 + (Man)3(GlcNAc)2 | 20.8 (0.8) | 20.9 (0.9) | 0.03 | 0.613 | 0.786 |
| C6 | GN4S | 6184.7 | 6184.7 | 9.8 | (Hex)5 (HexNAc)6 (Deoxyhexose)4 + (Man)3(GlcNAc)2 | 21.4 (0.7) | 21.5 (0.8) | 0.047 | 0.147 | 0.310 |
| C7 | G5N4F4 | 6232.8 | 6232.7 | 9.9 | (Hex)7 (HexNAc)6 (Deoxyhexose)2 + (Man)3(GlcNAc)2 | 23.8 (0.8) | 24.0 (1.0) | 0.129 | 0.004 | 0.028 |
| C8 | G7N4F2 | 6256.8 | 6256.7 | 9.7 | (Hex)4 (HexNAc)5 (Deoxyhexose)5 (NeuAc)1 + (Man)3(GlcNAc)2 | 20.4 (0.7) | 20.4 (0.7) | -0.074 | 0.177 | 0.357 |
| C9 | G4NF4 | 6263.8 | 6263.8 | 9.1 | (Hex)6 (HexNAc)1 (Deoxyhexose)2 (NeuAc)1 + (Man)3(GlcNAc)2 | 20.8 (0.8) | 21.0 (0.9) | 0.109 | 0.013 | 0.067 |
| C10 | G6-NF3S | 6278.0 | 6277.9 | 9.7 | (Hex)1 (HexNAc)4 (Deoxyhexose)2 + (Man)3(GlcNAc)2 | 19.7 (0.9) | 19.8 (0.8) | 0.049 | 0.315 | 0.531 |
| C11 | GN2F2 | 6290.8 | 6290.7 | 9.4 | (HexNAc)9 (Deoxyhexose)4 (NeuAc)1 + (Man)3(GlcNAc)2 | 19.4 (0.7) | 19.7 (0.7) | 0.181 | <0.001 | <0.001 |
| C12 | G9N2S | 6305.7 | 6305.7 | 9.5 | (Hex)6 (HexNAc)2 (Deoxyhexose)3 + (Man)3(GlcNAc)2 | 23.3 (0.9) | 23.6 (1.0) | 0.173 | 0.001 | 0.012 |
| C13 | G6F3 | 6377.8 | 6377.7 | 10.0 | (Hex)6 (HexNAc)4 (Deoxyhexose)5 (NeuAc)1 + (Man)3(GlcNAc)2 | 21.9 (0.9) | 22.1 (1.0) | 0.063 | 0.207 | 0.410 |
| C14 | G6N2F5S | 6401.8 | 6401.7 | 9.3 | (Hex)4 (HexNAc)5 (Deoxyhexose)4 (NeuAc)2 + (Man)3(GlcNAc)2 | 19.2 (0.8) | 19.3 (0.7) | -0.041 | 0.421 | 0.635 |
| C15 | G4N3F4S2 | 6435.8 | 6435.8 | 9.4 | (Hex)6 (HexNAc)5 (Deoxyhexose)6 + (Man)3(GlcNAc)2 | 18.9 (0.7) | 19.2 (0.8) | 0.201 | <0.001 | <0.001 |
| C16 | G6N3F6 | 6452.9 | 6452.8 | 9.7 | (Hex)2 (HexNAc)6 (Deoxyhexose)1 (NeuAc)1 + (Man)3(GlcNAc)2 | 21.1 (0.9) | 21.3 (1.0) | 0.084 | 0.132 | 0.301 |
| C17 | GN4F6 | 6523.8 | 6523.8 | 9.7 | (Hex)6 (HexNAc)4 (Deoxyhexose)6 (NeuAc)1 + (Man)3(GlcNAc)2 | 20.9 (0.8) | 21.0 (1.0) | 0.096 | 0.055 | 0.179 |
| C18 | G6N2F6S | 6597.9 | 6597.8 | 9.9 | (Hex)8 (HexNAc)7 (Deoxyhexose)2 + (Man)3(GlcNAc)2 | 22.1 (0.8) | 22.3 (1.0) | 0.154 | 0.001 | 0.014 |
| C19 | G7N6F2 | 6669.9 | 6669.8 | 9.7 | (Hex)6 (HexNAc)6 (Deoxyhexose)4 (NeuAc)1 + (Man)3(GlcNAc)2 | 19.9 (0.8) | 20.0 (0.9) | 0.04 | 0.328 | 0.531 |
| C20 | G4N3F5S | 6743.9 | 6743.9 | 10.1 | (Hex)9 (HexNAc)7 (Deoxyhexose)2 + (Man)3(GlcNAc)2 | 20.1 (0.9) | 20.3 (0.9) | 0.072 | 0.173 | 0.357 |
| C21 | G6N3F4S | 6890.0 | 6889.9 | 12.4 | (Hex)8 (HexNAc)7 (Deoxyhexose)4 + (Man)3(GlcNAc)2 | 20.1 (0.9) | 20.3 (1.0) | 0.133 | 0.015 | 0.071 |
